# Properties of non-coding DNA and identification of putative *cis*-regulatory elements in *Theileria parva*

**DOI:** 10.1186/1471-2164-9-582

**Published:** 2008-12-03

**Authors:** Xiang Guo, Joana C Silva

**Affiliations:** 1 The Institute for Genomic Research/J. Craig Venter Institute, 9712 Medical Center Drive, Rockville, MD 20850, USA; 2 Advanced Biomedical Computing Center, NCI-Frederick/SAIC-Frederick Inc., Frederick, MD 21702, USA; 3 Institute for Genome Sciences, University of Maryland School of Medicine, Baltimore, MD 21201, USA; 4 Department of Microbiology and Immunology, University of Maryland School of Medicine, Baltimore, MD 21201, USA

## Abstract

**Background:**

Parasites in the genus *Theileria *cause lymphoproliferative diseases in cattle, resulting in enormous socio-economic losses. The availability of the genome sequences and annotation for *T. parva *and *T. annulata *has facilitated the study of parasite biology and their relationship with host cell transformation and tropism. However, the mechanism of transcriptional regulation in this genus, which may be key to understanding fundamental aspects of its parasitology, remains poorly understood. In this study, we analyze the evolution of non-coding sequences in the *Theileria *genome and identify conserved sequence elements that may be involved in gene regulation of these parasitic species.

**Results:**

Intergenic regions and introns in *Theileria *are short, and their length distributions are considerably right-skewed. Intergenic regions flanked by genes in 5'-5' orientation tend to be longer and slightly more AT-rich than those flanked by two stop codons; intergenic regions flanked by genes in 3'-5' orientation have intermediate values of length and AT composition. Intron position is negatively correlated with intron length, and positively correlated with GC content. Using stringent criteria, we identified a set of high-quality orthologous non-coding sequences between *T. parva *and *T. annulata*, and determined the distribution of selective constraints across regions, which are shown to be higher close to translation start sites. A positive correlation between constraint and length in both intergenic regions and introns suggests a tight control over length expansion of non-coding regions. Genome-wide searches for functional elements revealed several conserved motifs in intergenic regions of *Theileria *genomes. Two such motifs are preferentially located within the first 60 base pairs upstream of transcription start sites in *T. parva*, are preferentially associated with specific protein functional categories, and have significant similarity to know regulatory motifs in other species. These results suggest that these two motifs are likely to represent transcription factor binding sites in *Theileria*.

**Conclusion:**

*Theileria *genomes are highly compact, with selection seemingly favoring short introns and intergenic regions. Three over-represented sequence motifs were independently identified in intergenic regions of both *Theileria *species, and the evidence suggests that at least two of them play a role in transcriptional control in *T. parva*. These are prime candidates for experimental validation of transcription factor binding sites in this single-celled eukaryotic parasite. Sequences similar to two of these *Theileria *motifs are conserved in *Plasmodium *hinting at the possibility of common regulatory machinery across the phylum *Apicomplexa*.

## Background

Species in the genus *Theileria *are the causative agents of lymphoproliferative diseases in cattle. The high mortality and morbidity in cattle associated with these diseases cause devastating socio-economic losses [1]. The species that cause the largest economic burden are *T. parva*, which causes East Coast fever, and *T. annulata*, which causes tropical theileriosis. Both species are transmitted by ticks and have complex intracellular life-cycles [2]. Two intracellular stages follow the introduction of sporozoite stage into mammalian hosts, the intra-lymphocytic schizont stage and the intra-erythrocytic piroplasm stage. The schizont stage has a unique ability to cause infected host cells to proliferate indefinitely, resulting in a leukemia-like phenotype [3]. Stage differentiation takes place at the time of parasite transmission to and from hosts, as well as during their establishment and amplification within the host. It has been shown that the molecular regulation of stage differentiation could be targeted for the development of new disease control strategies [4]. One such target are the regulators of gene expression, since they may determine the progression through life-cycle stages. However, little is known about the extent of, and the elements involved in, transcriptional regulation in these protozoan parasites.

*Theileria *species are among several thousand unicellular eukaryotic parasites in the phylum *Apicomplexa*, whose members include *Plasmodium falciparum*, the most deadly causative agent of malaria, and *Toxoplasma gondii*, one of the most successful parasites, infecting more than 30% of the human population and imposing a severe threat to immuno-compromised individuals. Apicomplexans are thought to have reduced transcriptional machinery relative to multicellular eukaryotes. The highly biased nucleotide composition of the apicomplexan genomes sequenced to date hamper the detection of *bona fide *regulatory elements [5], thus making it difficult to assess the extent of that reduction. Classical eukaryotic promoter elements such as the TATA-box and the CAAT-box appear to be absent in *T. gondii *and *P. falciparum *[6]. General transcription factors such as TFIIA and most of the TATA-binding protein associated factors are not found in apicomplexan genomes [7]. Instead, non-canonical regulatory motifs are correlated with gene expression in *T. gondii *[8]. However, recent studies that take into account the genome composition and/or the timing of gene expression are uncovering novel conserved sequence motifs, leading to a considerable expansion of the repertoire of known and putative transcription regulators in this phylum [9-14]. In *T. parva*, a massively parallel signature sequencing (MPSS) study indicates that the majority of genes are transcriptionally active in the schizont stage, but their transcription may not be stringently regulated and, instead, vary stochastically between different host cells or time points. Active regulation of transcription was observed only at certain classes of loci [15]. In *T. annulata*, mobility shift assays have identified an element upstream of the *TamS1 *gene that is a site of DNA-protein interactions during differentiation to the merozoite [16].

The complex life-cycle of *Theileria *poses a serious challenge to the development of a workable laboratory system, thus making it difficult to study the regulatory mechanism and developmental biology of this organism. Recently, the genome sequences of *T. parva *and *T. annulata *were published [17,18], enabling the study of the unique biological characteristics of *Theileria *parasites using systematic and global bioinformatics approaches. The genomes of *T. parva *and *T. annulata *are ~8 Mb long, with approximately 4000 genes each, identified through both automated and manual annotation. The gene density in these two genomes is fairly high, with the annotated coding sequences comprising ~70% of the nuclear DNA, introns ~10% and the remaining 20% consisting of intergenic regions (IGRs) [17,18]. The short length of *Theileria *IGRs, which average ~400 base pairs (bp), again suggests a different mechanism of transcriptional regulation from those found in multi-cellular eukaryotes. More importantly, these compact IGRs allow us to assess the feasibility of finding regulatory sequences with an exhaustive scan of the non-coding segment of the genome, and in the absence of comprehensive gene expression data.

In the current work, we try to understand the evolutionary forces that determine the characteristic of non-coding regions in *Theileria*, such as length, nucleotide composition and degree of conservation. Selective constraint is calculated based on alignment of orthologous IGRs and introns of *T. parva *and *T. annulata*. In particular, this study aimed to test whether sequence conservation in general, and conserved sequence motifs in particular, are most common in IGRs flanked by genes in head-to-head orientation, as expected if transcriptional regulation is an important component in the regulation of gene expression in *Theileria*. We apply a *de novo *motif discovery algorithm to identify putative *cis*-regulatory elements in IGRs and compare their conservation in two species. Candidate motifs are then characterized based on their location relative to transcription start sites and the function of neighboring genes in *T. parva*, their similarity to known transcription factor binding sites, and their distribution pattern in different non-coding genomic regions. In this first comprehensive study of non-coding sequence evolution and motif discovery in *Theileria*, we have demonstrated that selection favors short introns and IGRs, and identified conserved sequence motifs whose role in gene regulation can now be tested by experimental approaches.

## Results

### Characterization of non-coding DNA in *T. parva *and *T. annulata*

**Figure 1:**
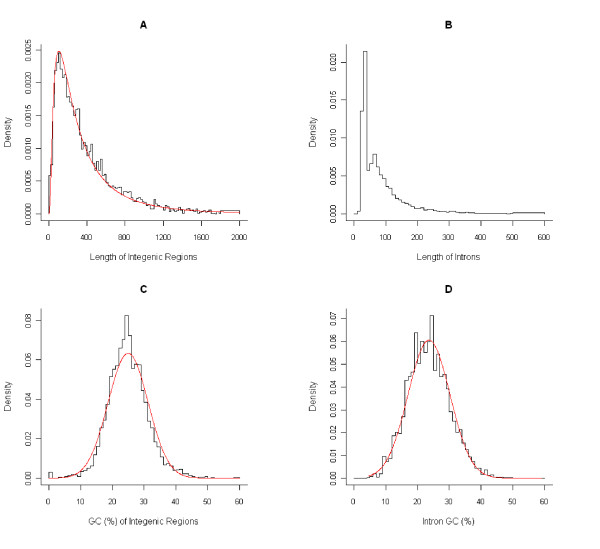
**Distribution of length and GC content of non-coding DNA in *T. parva***. Histograms of intergenic region length (A) and intron length (B), and GC content (%) of intergenic regions (C) and introns (D). The length of intergenic regions fits a lognormal distribution (A), while GC content of both types of non-coding DNA is normally distributed (C, D). The histogram of intron length is biomodal, which probably results from the overlay of two independent distributions of small (mode = 55) and large (mode = 80) introns, much like what had been documented for other organism [19]. Similar results are found in *T. annulata *(not shown).

The length and "guanine and cytosine" (GC) content of introns and IGRs are similarly distributed in *T. parva *(intron number = 10408; IGR number = 3982) and *T. annulata *(intron number = 10816; IGR number = 3738). IGR length fits a lognormal distribution with estimated modal value of 105 bp (Figure 1). The positive skew in the length distribution suggests that selective pressure maintains IGRs small, possibly to minimize genome size. Alternatively, such a distribution could result from the sporadic accumulation of large insertions in IGRs that are gradually eroded by more frequent deletions. The distribution of intron length is also right-skewed, but it is bimodal, similar to what is observed in other eukaryotes [19]. This suggests the presence of two distinct classes of introns: short introns, which make up the majority of the sample, with a modal value at 55 bp, and long introns, with a modal value of 80 bp. The distribution of GC content is approximately normal for both introns and IGRs (Figure 1). The average GC content across all IGRs is 26.2 ± 6.4% and 24.0 ± 6.5% in *T. parva *and *T. annulata*, respectively, while the average in introns is slightly lower (24.5 ± 6.6% GC in *T. parva *and 22.4 ± 6.9% GC in *T. annulata*). The GC content in non-coding regions is ~10% lower than the overall genome GC content in these species, which is approximately 34.1% in *T. parva *and 32.5% in *T. annulata *[17,18]. This difference suggests a mutational bias towards adenine and thymine (AT) and/or the accumulation of AT-rich regulatory motifs in non-coding regions of the genome.

In order to study the evolution of non-coding regions, homologous sequence segments between species are required. Establishing homology between non-coding regions is often difficult due to the lack of conserved landmarks that results from the accumulation of insertions, deletions and substitutions. In addition, the exact location of IGRs and introns is uncertain due to the probabilistic nature of the gene models generated through automated annotation. For this study, we identified a high-quality subset of introns and IGRs for which homology could be inferred with high probability (see Methods). The remaining analyses in this section were performed on this data subset, unless otherwise noted. The GC content in this subset of IGRs and introns (Table 1) is lower than the overall average, suggesting that coding regions that have not yet been identified are present in regions currently identified as non-coding.

**Table 1:** Property of orthologous intergenic regions (IGRs) and introns

	**Count**	**Length (bp)**	**GC (%)**	**Constraint (%)**
***T. parva *IGRs**	990	356.6 ± 9.8	23.3 ± 0.17	35.6 ± 0.59
***T. annulata *IGRs**	990	336.2 ± 9.1	21.9 ± 0.19	36.8 ± 0.57

***T. parva *Introns**	1487	75.6 ± 1.83	21.9 ± 0.16	28.4 ± 0.52
***T. annulata *Introns**	1487	63.8 ± 1.21	21.1 ± 0.16	30.3 ± 0.52

Each value of length, GC content and selective constraint is the mean ± SEM.

**Figure 2:**
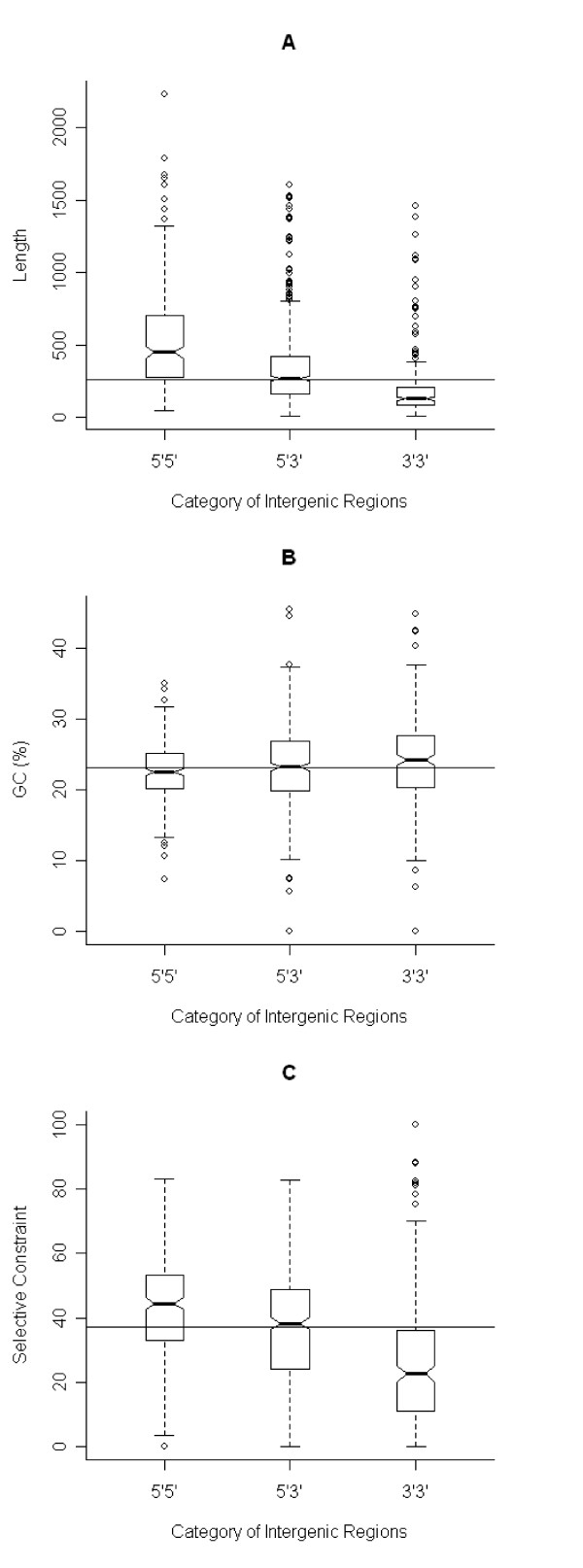
**Length, GC content and selective constraint distributions in three classes of intergenic regions in *T. parva***. Distribution of length (A), GC content (B) and selective constraint (C) per class of intergenic region (IGR) are depicted by boxplots. The three IGR classes (5'-5', 3'-5', 3'-3') are named according to the orientation of the flanking genes. Each box (interquartile range, IQR) contains the sample's 25% to 75% range (quartiles Q1 to Q3, respectively), with the bottleneck placed at the sample median. Horizontal tick marks show the range of all elements within Q1-1.5*IQR and Q3+1.5*IQR. Open circles mark data points outside this range, which are considered outliers. The width of the bottleneck (i.e the length of the V-shaped notch) is an indication of the confidence of the median; a lack of overlap of the bottleneck between samples implies that the medians are significantly different at ~95% confidence level. Similar results are found in *T. annulata *(not shown).

IGRs can be classified into three classes based on the orientation of the genes that flank them: 5'-5', 3'-3' or 5'-3', when surrounded respectively by two start codons, two termination codons or a start and a stop codon of successive genes. The length and GC content distributions differ significantly among IGR classes (*p *< 0.001, Kruskal-Wallis test). IGR length increases with increasing number of start codons (0, 1, or 2) flanking it (Figure 2A). This could be due to errors in determining the correct start codon, with the resulting gene models being consistently shorter than the true genes. This hypothesis seems unlikely, as it would result in IGRs enriched with coding segments, which in turn should increase GC content. In fact, the opposite trend in GC content is observed. In IGRs, GC content decreases with the number of flanking start codons, such that 5'-5' IGRs have the lowest %GC, 3'-3' IGRs have the highest %GC, and %GC of 3'-5' IGRs falls in between those of the other two classes (Figure 2B). A more likely explanation for the length trend among the three IGR classes is the preferential accumulation of functional motifs upstream of genes, causing 5'-5' IGR to be longest and 3'-3' IGRs to be the shortest. This hypothesis is supported by the significant difference in selective constraint across IGR classes (see below).

**Figure 3:**
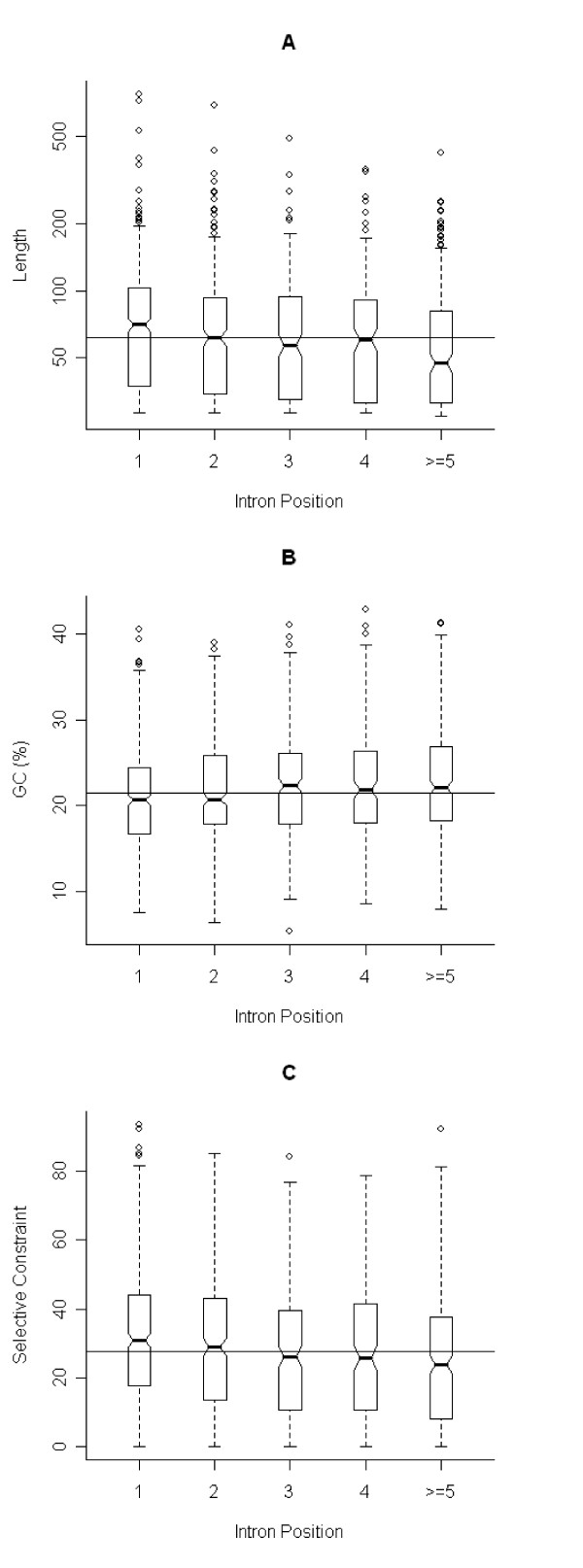
**Length, GC content and selective constraint distributions across intron positions in *T. parva***. Distribution of length (A), GC content (B) and selective constraint (C) in introns of different ordinal numbers are depicted by boxplots. The last class averages across introns of position equal to or larger than 5. Graph description as in legend of Figure 2. In the boxplot of length distribution, a log scale is used for the vertical axis since intron lengths span a large range. Similar results are found in *T. annulata *(not shown).

Intron length has been shown to be negatively correlated to intron ordinal number in *Arabidopsis *and in murids [20,21], a pattern that can be attributed to the uneven distribution of functional DNA elements across introns, with larger fractions present towards the 5' end of genes. A similar relationship is observed in *Theileria*, with the average length for first introns being 108 bp, and that value gradually decreasing to 94 bp, 92 bp, 87 bp and 84 bp for intron ordinal number 2, 3, 4 and ≥ 5, respectively (Figure 3A). In addition, GC content is positively related with intron position (Figure 3B). Both intron length and GC content differ significantly among different classes of introns (*p *< 0.001, Kruskal-Wallis test).

The degree of selective constraint was estimated for each high-quality, orthologous intron pair (*n *= 1487) and IGR pair (*n *= 990), based on individual global alignments. Our results show the degree of conservation to be higher in IGRs than in introns (Table 1). Interestingly, selective constraint in IGRs of *T. parva *increases with the number of start codons that flank it (Figure 2C), and decreases with intron ordinal number (Figure 3C). The difference is statistically significant among different classes of IGRs and among introns (*p *< 0.001, Kruskal-Wallis test). These results point to the accumulation of functional motifs upstream of genes and also in introns that are closer to the 5' end of genes, and are consistent with the assertion of a direct relationship between the length of non-coding regions and the frequency of functional motifs.

### Conserved Sequence Motifs and their Biological Relevance

**Figure 4:**
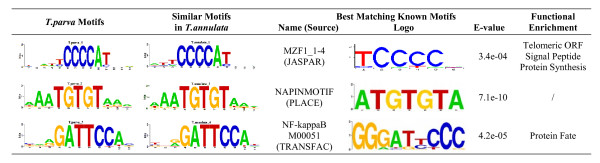
**Conserved motifs and their best matches in databases of known motifs**. Pictogram representation of the top three MEME-derived motifs in *T. parva *and their most similar motifs in *T. annulata *(two left columns). The best matches to conserved motifs in *T. parva *among known motifs are shown (center), including name, database source, sequence logo, and STAMP E-value, which is a relative measure of similarity between two motifs based on simulated position specific score matrix models. Functional and structural annotations enriched in downstream genes of each *T. parva *motif are shown on the right.

Using MEME, we mined the 300 nucleotides proximal to the 5' end of all genes in either *T. parva *or *T. annulata *for enriched motifs. Given the average IGR length of ~400 bp, this length restriction used in the initial motif search should adequately capture relevant regulatory regions, while at the same time minimizing the probability of motifs affecting neighboring genes. Table 2 lists the top five statistically significant motifs found in each species. Motifs 1, 2, 3 in *T. parva *and motifs 1, 2, 4 in *T. annulata *are found in hundreds of copies per genome in the upstream region of genes, while the frequency of other two motifs is an order of magnitude lower (Table 2). Motif comparison indicates that top three motifs in *T. parva *are almost identical to motifs 1, 2 and 4 in *T. annulata *(Figure 4). We further investigated the biological relevance of these three conserved motifs using transcription start site (TSS) and gene functional annotation information.

**Table 2:** Top five motifs in 5' intergenic regions of *T. parva *and *T. annulata*

	**Consensus**	**Width**	**Bits**	**E-value**	**Sites**
***T. parva *Motif 1**	HDWTYCCCCATVVR	14	16.0	5.7e-472	639
**2**	WAATGTGTARR	11	13.6	5.2e-414	1199
**3**	BDRGATTCCAY	11	16.0	8.5e-108	298
**4**	CCBCBRSARGGAGCY	15	24.2	3.7e-048	39
**5**	GCTCCCKCYWGGSG	14	26.2	1.2e-019	19

***T. annulata *Motif 1**	WTTCCCCATNND	12	15.5	4.1e-388	615
**2**	WAATGTGTAAW	11	13.3	1.1e-348	1173
**3**	TGTTTGTTCTGATGG	15	27.2	7.1e-140	56
**4**	BDRGATTCCAY	11	15.8	8.8e-103	302
**5**	GTAATATTTCTGTAA	15	23.7	5.0e-096	62

Motifs are identified by MEME and their consensus sequences are shown here using IUPAC (International union of pure and applied chemistry) nucleic acid codes. Information content in bits is calculated based on the first-order Markov background model. The E-value estimates the number of motifs with the same width and number of occurrence that would have equal or higher likelihood in the same number of random sequences generated by the background model. Sites refer to the number of sequences that MEME used to build each motif model.

**Figure 5:**
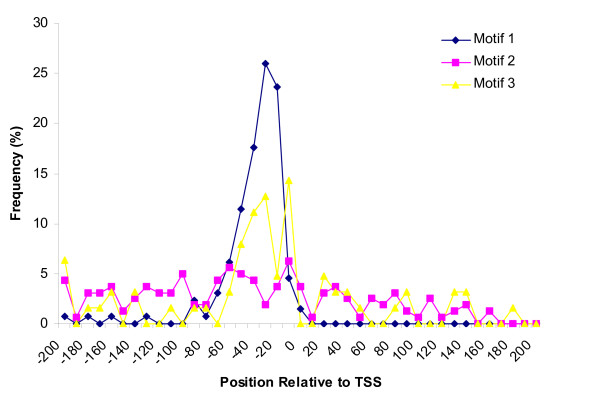
**Distribution of conserved sequence motifs with respect to putative transcription start sites in *T. parva***. The distances between the first base of each motif and the TSS were determined for all genes for which both types of information were available. Distances are binned in 10-bp intervals. The frequency is determined based on 132 sites for motif 1, 168 sites for motif 2, and 67 sites for motif 3.

First, we looked for an association between putative motifs and the transcriptional process by determining whether they had a fixed position relative to the TSS [22]. Putative TSSs have been determined for 650 *T. parva *genes based on EST mapping, and on average map 138 nucleotides upstream of the start codon. *T. parva *motifs 1, 2 and 3 were present in IGRs next to, respectively, 132, 168 and 67 of the 650 genes with a defined UTR. Figure 5 shows the distribution of three motifs relative to the TSS. The distribution of motif 1 is remarkably restricted, with motifs almost exclusively located within 60 bases upstream of the TSS. Motif 3 is also enriched in this region, with more than 50% of the sequences located within this 60 bp range. However, the distribution of motif 2 is relatively flat, with ~70% of the sequences located upstream of the TSS and the remaining 30% located between the TSS and the initiation codon (Figure 5).

Next, we investigated the biological relevance for each motif in the context of the function of adjacent genes. A hypergeometric test demonstrated that certain functional categories are significantly enriched among genes downstream of motifs 1 and 3 (*p *< 0.01), but not of motif 2. Motif 1 is associated with genes involved in protein synthesis, with telomeric ORFs, and with proteins containing signal peptides, while motif 3 is associated with genes related to protein fate (Additional file 1). Using STAMP [23], we identified known motifs that are most similar to each of the three *Theileria *motifs. Motif 1 has similarity to a DNA consensus binding site for myeloid zinc finger protein 1 (MZF1), a C2H2 zinc finger transcription factor involved in granulopoiesis, cellular proliferation and oncogenesis [24] (Figure 4). In addition, motif 1 is identical to the motif ATGGGGC, which has been identified independently in different studies in *Plasmodium *[11,14,25] and which may be preferentially associated with metabolic genes that are highly expressed during the trophozoite stage [14]. For motif 2, STAMP detected a highly similar sequence element that is known to interact with a nuclear protein in a plant species [26] (Figure 4). It is also nearly identical with a *P. falciparum *conserved motif TGTGT(G/A)(A/T) which, much like motif 2, has a widespread genome distribution [14]. Motif 3 has significant similarity to the binding site for NF-κB, a family of transcription factors whose activation has been shown to be associated with host invasion in various pathogens, including *T. parva *[27] (Figure 4).

**Figure 6:**
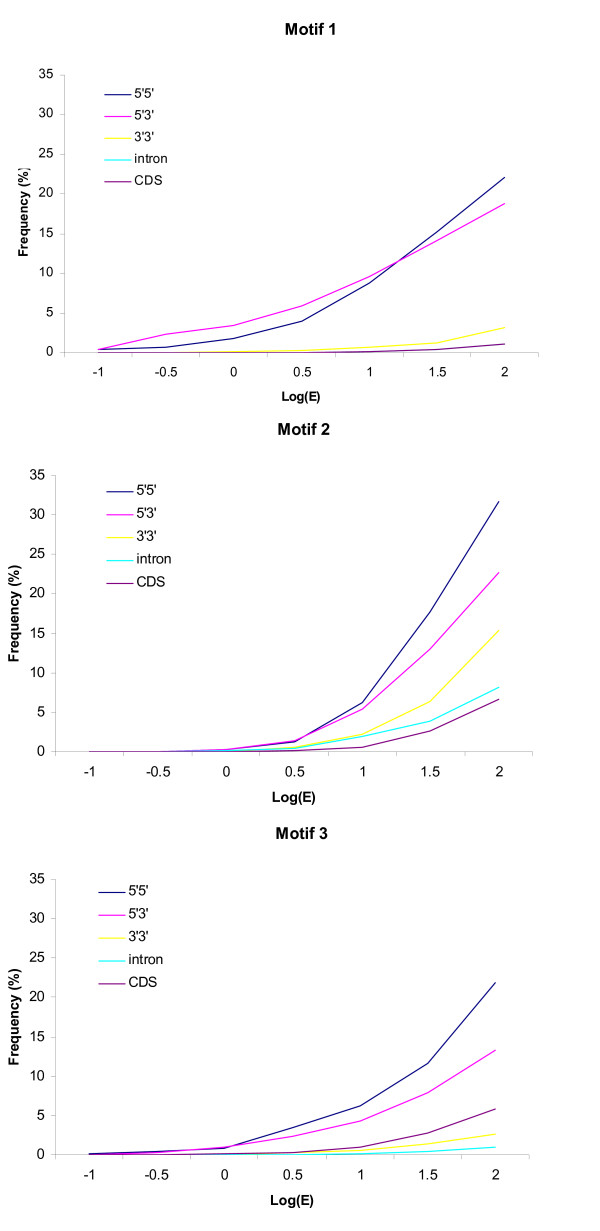
**Distribution of three conserved motifs in different partition of the *T. parva *genome**. The distribution of the three highest-scoring MEME-derived motifs in *T. parva *was determined in coding regions (CDS), 5'-5', 5'-3' and 3'-3' intergenic regions, and introns using the MAST algorithm. Relative frequency of each type of sequence with at least one occurrence of a motif is plotted as a function of the MAST E-value cutoff.

Finally, we investigated the distribution pattern of three motifs across the entire *T. parva *genome. MAST was used to detect occurrence of each motif in coding regions (CDS), introns, and the three types of IGRs, at different probability thresholds. All three motifs were found more often in 5'-5' and 5'-3' IGRs than in the other regions (Figure 6). Together with the higher selective constraints observed in IGRs bordered by 5' ends of genes, these results are consistent with the presence of regulatory motifs in the upstream region of genes. Although the average length of CDS (1407 bp) is much longer than that of IGRs (407 bp), the frequency of the three motifs is higher in 5'-5' and 3'-5' IGRs than in CDS (Figure 6). This distribution pattern provides an additional evidence for the biological significance of our putative motifs. In addition to its presence in IGRs flanked by at least one start codon, motif 2 is also found in 3'-3' IGRs and in introns, revealing a widespread distribution throughout non-coding regions of the *T. parva *genome.

## Discussion

The fraction of non-coding DNA in the majority of bacterial and archaeal genomes is between 6% and 14%, but close to 90% in multicellular eukaryotes [28]. Single-celled eukaryotes have a higher proportion of non-coding DNA than prokaryotes, but a much more compact genome than multicellular eukaryotes. The smallest known nuclear genome, that of a chlorarachniophyte nucleomorph, contains 22% intergenic DNA [29], and the single-celled model organism *Saccharomyces cerevisiae*, the budding yeast, contains 30% intergenic DNA [30]. Intron length and number varies among taxa, and dramatic difference can be seen across related species. Approximately 40% of genes contain introns in the fission yeast, *Schizosaccharomyces pombe*, while only 4% of genes have introns in *S. cerevisiae *[30]. The non-protein coding regions of multi-cellular eukaryotes include the remnants of transposable elements that have lost functionality, ribosomal genes, motifs involved in gene regulation and chromosomal structure and possibly other unknown functions. Only a few eukaryotic unicellular parasites have so far been shown to contain transposable elements [31-34], and many of these organisms are known to lack transposable elements all together [17,35,36], which probably explains much of the observed difference in the amount of non-coding DNA between these organisms and other eukaryotes. However, non-coding regions of these small parasitic genomes remain remarkably understudied, and little is known about the forces that shape them.

The genome of *Theileria *species is highly compact. Non-coding sequences make up ~30% of the genome, as the average length of both introns and IGRs is smaller than that of many eukaryote genomes, and no transposable elements have so far been found. Our results show that a large fraction of non-coding DNA is kept constant due to purifying selection (Table 1). This high conservation rate confirms the functional importance of non-coding sequences in *Theileria*, which goes beyond a role as passive intergenic spacers. This assertion is further supported by the higher degree of sequence conservation in IGRs that border the 5' end of genes relative to what is observed in IGRs flanked by termination codons, since IGR sequence conservation between species in regions upstream of genes is associated with the presence of regulatory elements [37]. The presence of functionally important motifs at higher frequencies in 5'-5' IGRs relative to 3'-3' IGRs limits the fixation of deletion events in the former regions, which in turn remain longer.

Introns play an important role in gene transcription regulation and mRNA processing, and functional elements are often found in first introns [38,39]. First introns tend to be longer and more conserved than introns of higher ordinal number, a pattern observed both in mammals [21] and in invertebrates [40]. Our analyses yielded a similar result in *Theileria*, extending this pattern to unicellular eukaryotes for the first time. These results also suggest the accumulation of functional elements towards the 5' end of *Theileria *genes.

The study of transcriptional regulation in apicomplexan parasites has identified some unique features of this large group of protists. Canonical elements seem to be absent from promoter regions, while non-canonical regulatory elements in upstream regions have been found to be involved in the regulation of gene expression. Transcriptome analysis has demonstrated active regulation of transcription in *T. parva *[15], but no regulatory elements or transcription factors have been identified so far.

In this study, we found three putative motifs that are present in hundreds of copies throughout the genome. Two of them, motifs 1 and 3 in *T. parva*, are preferentially located in the 60 nucleotides upstream of TSS, suggesting that they may be transcription factor binding sites in this species. Motif 1 appears to be enriched near telomere-associated ORFs and signal peptide-containing proteins. While the function of telomeric ORFs in *Theileria*, so called due to their extreme proximity to telomeres, remains unknown they encode hyper-polymorphic gene families [41]. In *Plasmodium*, gene families with these characteristics are known to be important to pathogenesis and antigenic variation [42,43]. In fact, a large fraction of *T. parva*'s telomeric ORFs expressed in the schizont stage contain predicted signal sequences, consistent with their involvement in host-parasite interaction [15]. The current discovery of a putative regulatory element preferentially located in upstream regions of telomeric ORFs may help the functional study and design of molecular tools to manipulate this important group of proteins in *Theileria*. Based on a MIPS classification of *T. parva *proteins [15], we detected an association of this motif with genes involved in protein synthesis, and database searches indicated that it is similar to a DNA consensus binding site for myeloid zinc finger protein 1 (MZF1), a C2H2 zinc finger transcription factor involved in cellular proliferation and oncogenesis [24]. The finding of an identical motif in *Plasmodium *associated with genes that are highly expressed during phases of rapid cellular multiplication makes it tempting to speculate whether motif 1 may be the binding site for an unidentified transcription factor in *Theileria *associated with protein synthesis and/or cell division.

Motif 3 appears to be associated with genes that are involved in protein modification, stabilization, degradation, targeting, sorting, translocation, and other protein fate-related functions. It is similar to the binding site for NF-κB transcription factors, which has been shown to be involved in host cell transformation mechanisms in various pathogens, including *Theileria *[27]. The schizont stage of *T. parva *induces sustained activation of NF-κB, which regulates the expression of genes involved in immune and inflammatory responses [44], proliferation [45], and survival [46]. Pathogens may also utilize the NF-κB system to enhance their own replication, survival, and dissemination within the host [47]. For example, NF-κB binding sites have been found in the enhancer region of the long terminal repeat [48] and 5' UTR [49] of human immunodeficiency virus (HIV), through which host NF-κB promotes viral replication and survival. Although no homolog to human NF-κB has been identified in *Theileria*, it is conceivable that a transcription factor with a similar binding motif to that used by NF-κB might exist in these species. To that effect we searched the *T. parva *protein-coding genes for peptides with homology to the IPT domain of NF-κB, which is involved in DNA binding. Our BLASTP analysis uncovered a significant match (*E *= 3.7e-5) to a hypothetical protein, TP02-0125, which has 33% identity and 55% similarity to the DNA-binding domain of NF-κB. A support vector machine-based algorithm predicted TP02-0125 to be a DNA-binding protein [50]. This result, together with the distribution characteristics of motif 3, suggests that attempting the experimental validation of this motif is warranted.

In contrast with the previous two motifs, motif 2 is found throughout non-coding regions, without a localized distribution relative to TSS or specific protein functional classes. However, an almost identical motif has been demonstrated to interact with a nuclear protein in developing rape seeds [18], suggesting the role of our motif as a binding site for regulatory proteins other than transcription factors. Interestingly, our motif is highly similar to a conserved motif in *P. falciparum *that also has a widespread genome distribution [14,51]. While it is possible that this sequence pattern is a characteristic feature of apicomplexan genomes, its function remains unknown.

As more *Theileria *genome sequences become available, the search for conserved motifs in non-coding sequences will have added power. A comprehensive list of conserved elements may be derived by combining phylogenetic footprinting and *de novo *pattern matching algorithms. Various experimental approaches are available for the verification of putative regulatory elements, including *in vitro *protein binding experiments, *in vivo *DNA structure assays, and reverse genetics methods. In particular electrophoretic mobility shift essays have been documented to work well in apicomplexan systems [9,11,16]. Revealing the functional potential of these conserved elements will advance the study of gene regulation in *Theileria *and possibly lead to the improved control and therapeutics for East Coast Fever and tropical therileriosis in cattle.

## Conclusion

The highly compact genome of *Theileria *seems to result from selection pressure for small introns and IGRs. While, much like in other apicomplexan genomes, classical eukaryotic promoter elements have not been found in *Theileria*, genome-wide *de novo *searches identified several conserved sequence motifs in IGRs. Two putative *T. parva *motifs have localized distribution relative to transcriptional start sites and are preferentially associated with specific protein functions, which is consistent with the hypothesis that they participate in transcriptional regulation in this eukaryotic parasite. The fact that conserved motifs with similar sequence are found in *Plasmodium *hints at the possibility of common regulatory mechanisms across the phylum *Apicomplexa*.

## Methods

### Sequence Data

An in-house database was developed to store and analyze genome sequences and annotations of two *Theileria *species. There are 4011 annotated genes in *T. parva*, with 10408 introns. 5' and 3' UTR information is available for 650 and 545 genes, respectively. The total number of IGRs, defined as complete DNA sequences between start and/or stop codons of two consecutive annotated genes, is 3982 in *T. parva. T. annulata *has 3784 annotated genes, with 10816 introns and a total of 3738 IGRs. Transfer and ribosomal RNA genes were also excluded from IGRs.

### Homology Identification

Orthologous clusters (OCs) were created using the publicly available gene annotations of six apicomplexan genomes, namely *T. parva, T. annulata, Babesia bovis, Plasmodium falciparum *(version released with PlasmoDB 5.0 Beta), *Plasmodium yoelii*, and *Cryptosporidium parvum*. Except for *P. falciparum*, the original genome annotation release was used for each species. Jaccard-filtered OC analysis [52] was used to construct the final ortholog set, and resulted in 3137 OCs containing at least one gene from each of the two *Theileria *species (Jaccard filter cutoff at 0.6). OCs with paralogs in *Theileria *were excluded, and 2904 OCs were retained that include exactly one gene from each species. OCs were also excluded if the ratio of the shortest to the longest of the two *Theileria *genes was smaller than 0.9. Genes eliminated by this criterion are likely to have an incorrect structural gene annotation in one or both species, or to contain repeats that render sequence alignment questionable. The final set of high-quality OCs contains 1956 genes, with one sequence each in *T. parva *and in *T. annulata*.

A high-quality (HQ) set of orthologous non-coding regions was then defined as follows: HQ orthologous IGRs are flanked by HQ OCs at both ends. HQ orthologous introns are those from HQ OCs with the same number of introns in both species; in addition, HQ orthologous introns are flanked by exon regions in which amino acid similarity is ≥ 75% between species. This cutoff was determined empirically and resulted in the elimination of 25% of introns. The final dataset contains 990 pairs of HQ orthologous IGRs and 1487 pairs of HQ orthologous introns.

### Selective Constraint

Orthologous non-coding sequences were aligned using *Owen *[53], to generate global alignments consisting of segments with significant sequence similarity (*p *< 0.001), also called hits, interspersed with segments for which sequence similarity was below that significance threshold. Selective constraint, *c*, in IGRs and introns was estimated according to *Shabalina *and *Kondrashov *[54]. It is defined as the fraction of invariant nucleotides within a sequence segment. Given a similarity (*s*) within a hit defined as the number of matches divided by the length of the shorter sequence (*l*_*short*_), and the probability *r *that a site is identical between two sequences due to non-deterministic reasons, selective constraint within a hit is estimated as $c_{short}=\frac{s-r}{1-r}$ for shorter sequence and $c_{long}=\frac{l_{short}}{l_{long}}*\frac{s-r}{1-r}$ for longer sequence. In this study, we estimated *r *from the similarity in third codon positions of 4-fold degenerate amino acids in HQ OCs between the two *Therileria *species; these should correspond mostly to sites that are identical due to chance or because not enough time has elapsed since the two species split for substitutions to occur. For an IGR or intron having *n *hits, the selective constraint is defined as the sum, over all *n *hits, of the total number of constrained nucleotides divided by the length *L *of non-coding sequence in either species, $\frac{\sum l_{hit}c_{hit}}{L}$[55].

### Motif Discovery

Although computational motif discovery approaches have traditionally been used to find over-represented patterns among co-regulated genes, they may also be applied to large sets of unrelated promoter regions [56]. In the current study, we retrieved up to 300 bases of IGRs flanking the translation start sites of all *T. parva *and *T. annulata *genes and used MEME (Multiple EM for Motif Elicitation) to search for over-represented motifs on both DNA strands. The search was done separately for each species. MEME looks for conserved ungapped blocks in a group of sequences using an iterative expectation-maximization algorithm [57]. A background model is used by MEME to calculate the log likelihood ratio and statistical significance of a motif. The model used in our search is a first-order Markov chain derived from all intergenic sequences in *T. parva *and *T. annulata *respectively. We assume the presence of either zero or one motif per sequence, with motif width between 5–15 bp. Putative motifs found in two species were compared by the Smith-Waterman local alignment method with similarity score defined by Pearson's correlation coefficient [23].

### Biological Relevance of Putative Motifs

Putative motifs were further investigated in terms of their location distribution in relation to TSS, functional characterization of downstream genes, similarity to known transcription factor binding sites, and occurrence pattern in different non-coding genomic regions. These four analyses were performed in *T. parva *only. First, we retrieved all *T. parva *genes with putative TSSs information available and looked at the intersection between these genes and our set of putative motifs. Distance between the first base of a motif and TSS was calculated and binned in 10-bp intervals. Localized distribution of motifs in relation to TSS will provide evidence for the biological relevance of putative motifs.

Secondly, we investigated if any specific gene function(s) is over-represented in the region downstream of each motif. *T. parva *genes have been previously annotated based on the Munich Information Centre for Protein Sequences (MIPS) catalog and Signal P software had been used to predict the existence of signal peptides/signal anchors in expressed genes [15]. We tested functional over-representation in terms of 9 MIPS categories, telomere association and signal peptide/signal anchor information associated with the group of genes whose 5' IGRs contain a particular motif. The statistical significance is measured by a test for hypergeometric distribution (Fisher's exact test). If *R *genes have been annotated with an annotation category and the total number of unique genes in the genome is *N*, then the *p*-value of finding at least *t *genes with the annotation category among *m *genes with the motif of interest in their upstream regions is:

$$P(t)=\sum_{i=t}^{\min(R,m)}\frac{\left(\begin{array}{l} R\\ i \end{array}\right)\left(\begin{array}{l} N-R\\ m-i \end{array}\right)}{\left(\begin{array}{l} N\\ m \end{array}\right)}$$

As multiple functional catagories were tested simultaneously, *p*-values are corrected for multiple hypotheses testing using a Bonferroni approach. The calculation was done using the function *phyper *in the statistical language R.

In the third analysis, we identified known motifs with significant similarity to our putative motifs using the web tool STAMP [23]. *T. parva *motifs were searched against a collection of databases including two comprehensive eukaryotic motif databases JASPAR [58] and TRANSFAC [59], drosophila DNase I footprint database FlyReg [60], plant motif databases PLACE [61] and AthaMap [62], prokaryotic motif databases RegTransBase [63] and DPInteract [64], as well as yeast motifs predicted by Harbison et al [65] and MacIsaac et al [66]. Motif similarity was estimated by the Smith-Waterman local alignment method with similarity score defined by Pearson's correlation coefficient. The significance is estimated based on simulated position specific scoring matrix (PSSM) models [67].

Lastly, we used MAST (Motif Alignment and Search Tool) to find all occurrences of each putative motif in *T. parva *non-coding regions [68]. The E-value of a match in an IGR or intron to a motif is based on a random sequence model derived from all non-coding sequences in *T. parva*. Various E-value cutoffs are used to retrieve matches with different statistical significance.

## Authors' contributions

JS conceived the project. JS and XG devised computational approaches, XG performed the analyses. XG and JS wrote the manuscript.

## Supplementary Material

Additional file 1 **Enrichment analysis of functional and structural annotations in *T. parva*.** The data provided represent the analysis of functional and structural enrichment of genes downstream of each putative motif in *T. parva*. Click here for file
