# Supplementary material for: Properties of non-coding DNA and identification of putative cis-regulatory elements in Theileria parva
Source: BMC Genomics. 2008 Dec 3;9:582. doi: 10.1186/1471-2164-9-582 (PMC2612703; doi:10.1186/1471-2164-9-582)
Supplement: Additional file 1 — Enrichment analysis of functional and structural annotations in T. parva. The data provided represent the analysis of functional and structural enrichment of genes downstream of each putative motif in T. parva. [file 1471-2164-9-582-S1.doc]

# Additional files

### Additional file 1. Enrichment analysis of functional and structural annotations.

| **Functional Category** | **# genes Category** | **Motif 1** | | | **Motif 2** | | | **Motif 3** | | |
| --- | --- | --- | --- | --- | --- | --- | --- | --- | --- | --- |
|  |  | **# genes assigned** | **p-value** | **corrected p-value** | **# genes assigned** | **p-value** | **corrected p-value** | **# genes assigned** | **p-value** | **corrected p-value** |
| Cell Cycle - DNA Processing | 107 | 9 | 0.98 | > 1 | 42 | 0.012 | 0.14 | 10 | 0.16 | > 1 |
| Cell Surface | 13 | 4 | 0.042 | 0.5 | 4 | 0.34 | > 1 | 0 | 0.63 | > 1 |
| Cellular Communication - Signal Transduction | 67 | 11 | 0.37 | > 1 | 24 | 0.11 | > 1 | 4 | 0.56 | > 1 |
| Cellular Transport and Transport Mechanism | 77 | 9 | 0.80 | > 1 | 21 | 0.63 | > 1 | 4 | 0.68 | > 1 |
| Metabolism – Energy | 182 | 34 | 0.12 | > 1 | 46 | 0.90 | > 1 | 20 | 0.025 | 0.3 |
| Protein Fate | 158 | 26 | 0.36 | > 1 | 45 | 0.60 | > 1 | 23 | 4.5E-4 | 5.4E-3 |
| Protein Synthesis | 204 | 70 | 6.0E-12 | 7.2E-11 | 52 | 0.90 | > 1 | 25 | 3.7E-3 | 0.044 |
| Telomeric ORF | 131 | 89 | 1.3E-43 | 1.6E-42 | 16 | 1.0 | > 1 | 1 | 1.0 | > 1 |
| Transcription | 127 | 30 | 7.2E-3 | 0.09 | 18 | 1.0 | > 1 | 8 | 0.60 | > 1 |
| Transport Facilitation | 49 | 9 | 0.24 | > 1 | 12 | 0.74 | > 1 | 3 | 0.49 | > 1 |
| Signal Peptide | 526 | 162 | 3.3E-21 | 4.0E-20 | 170 | 0.074 | 0.89 | 43 | 0.20 | > 1 |
| Signal Anchor | 200 | 29 | 0.66 | > 1 | 60 | 0.43 | > 1 | 7 | 0.98 | > 1 |

This table contains MIPS categories, telomeric ORF, signal peptide/anchor enrichment results for three sets of genes that contain motif 1, 2 or 3 in their upstream non-coding region respectively. The total number of genes is 4011, and the number of genes containing motifs 1, 2 or 3 in their 5’ regions are 639, 1199, and 298. Statistical significance was computed based on hypergeometric distribution with Bonferronni multiple hypothesis correction. The first column shows names of functional and structural annotations. The second column contains the total number of genes of each annotation for the whole genome. The third column contains for each annotation total number of genes that also contain motif 1 in their upstream non-coding region. The fourth column contains corrected p-values for the enrichment. Similarly, other columns show number of genes that are assigned to an annotation category and contain motif 2 or 3 in their upstream region, in addition to corrected p-values for the enrichment.
